# Medical Machiavellianism: the tradeoff between benefit and harm with targeted chemotherapy

**DOI:** 10.18632/oncotarget.6984

**Published:** 2016-01-22

**Authors:** Bryan Oronsky, Corey Carter, Anna Scicinska, Arnold Oronsky, Neil Oronsky, Michelle Lybeck, Jan Scicinski

**Affiliations:** ^1^ EpicentRx, Inc., Mountain View, CA, USA; ^2^ Walter Reed National Military Medical Center, Bethesda, MD, USA; ^3^ InterWest Partners, Menlo Park, CA, USA; ^4^ CFLS, LLC, San Jose, CA, USA

**Keywords:** cancer, adverse events, risk to benefit, medical machiavellianism

## Abstract

Machiavellianism is a word synonymous with the phrase “the end justifies the means”, and in this article we have coined the term Medical Machiavellianism to describe the ‘cruel-to-be-kind’ administration of toxic chemotherapeutic agents in apparent violation of the precept first do no harm, while acknowledging the ‘dirty hands’ dilemma of having to decide between and choose the lesser of two evils in the setting of advanced cancer—i.e. to treat or not to treat. The perception that ‘targeted’ therapies are relatively non-toxic and therefore respect the Hippocratic First Commandment by virtue of their narrow selectivity is belied by their often inherent promiscuity, addressing multiple targets either inadvertently or deliberately, which may result in multiple side effects.

The remarkable success of immunotherapy may have taken the bloom off the ‘targeted agent’ rose, however due to a lack of other approved treatment alternatives the toxicity of these agents may be overlooked or, at least, undervalued, especially given that the official measure of treatment success in oncology is overall survival (OS), not quality-of-life improvements.

By analogy with the MACH-IV personality survey (1970), [1] which measures high and low Machiavellian orientation, we have defined in this article a rudimentary MACH scale for selected targeted chemotherapies, based on the means-to-ends ratio of toxicity and benefit. It is our hope that this comparison between targeted agents will itself function as a means to an end—to help oncologists strike the right balance between efficacy, toxicity and quality of life in the management of their patients.

## INTRODUCTION

Irrespective of the clinical scenario the guiding principles of medical treatment are “primum non nocere” (“first do no harm”) and “primum succurrere” (“first hasten to help”) although in the management of cancer under the rubric of disease eradication the severe toxicities of potentially life-saving chemotherapies may and in fact usually do constitute an acceptable level of harm in apparent violation of the First Golden Rule.

It is a uniformly accepted oncologic tenet that, whatever the prognosis, even if the intent is only palliative, overall survival matters above all else [2, 3]. In other words, from the perspective of the medical establishment, for the sake of an overridingly just goal, namely, to postpone the existential threat of cancer, the end justifies the means. This in essence is a form of Medical Machiavellianism (MM), an epithet that originates from the name of Niccolo Machiavelli, the author of the 1513 political treatise, *Il Principe*, The Prince. This work, a handbook for despots and tyrants on the use of deceptive, manipulative, and ruthless self-serving practices to gain, hold, and expand power, is clearly antithetical to the Hippocratic tradition of beneficent and unselfish concern for the rights and welfare of patients. For Machiavelli “by any means necessary” was a morally permissible and practically expedient formula because the only virtue is success, no matter the methods necessary to achieve it. Taking inspiration from the work of Richard Christie and Florence Geis [1], who co-opted the term Machiavellianism, a byword for sneakiness, ambition, ruthlessness, and the open espousal of ‘playing hardball’, to describe a constellation of personality traits from which the MACH-IV personality scale was developed in 1970, we have similarly applied the Machiavelli premise of “by any means necessary” to highlight the tradeoffs between activity and adverse events in the treatment of cancer. However, as previously mentioned, Machiavellianism in its original intent is associated with deception, while the practice of medicine, in accordance with the Hippocratic oath and the Hippocratic tradition, should contain no element of deception. In this non-maleficent medical context, since physicians are honor and duty-bound to protect and serve the patient to the exclusion of third-party interests, including their own, MM, rather than implying any element of sham or deceit, is a therapeutic doctrine consistent with “I must be cruel to be kind” and “if it works, use it” because the benefit of overall survival balances or even outweighs considerations related to quality of life and/or cost.

Survival is the name of the game and in the evolving gambit-counter-gambit-counter-counter-gambit chess match of palliative cancer treatment where the oncologist selects a therapy and tumors ‘choose’ an adaptive strategy the sacrifice pawn is invariably quality of life even when the only objective, the best case scenario, is to force a stalemate from an otherwise desperate and hopeless situation. Nevertheless in reaction to the broad toxicities of conventional chemotherapy therapeutic strategies have evolved to only target cancer cells while sparing their normal counterparts (in what might be called somewhat facetiously primum non chemothere). However, even in this era of personalized medicine, where particular therapies are designed and developed for particular patients, based on a pharmacogenomic likelihood of benefit, the balance of antitumor activity versus toxicity is—or should be—a critical consideration.

While the applicability of this personalization on a general level remains at best an open question, it should be noted that the term “targeted” therapy is largely a misnomer since the most active molecular therapeutics (e.g. small molecule tyrosine kinase inhibitors [4] are more promiscuous than selective with multiple confirmed molecular targets (e.g. imatinib). And, in some cases, unintended ‘off-target’ effects may be clinically significant, such as inhibition of vascular endothelial growth factor receptor 2 (VEGFR2) with regorafenib [5].

The equation, ‘drug + molecular target = targeted therapy’, with its implication of narrow biological specificity and toxicity, almost certainly oversimplifies the enormously complex and coordinated interactions that occur *in vivo* [5]. The analogy of Ehrlich's magic bullet applies to targeted therapies but only in the sense of a ricocheting bullet simultaneously hitting multiple targets.

The term targeted therapy also implies, incorrectly, that conventional chemotherapy is untargeted. In fact, all clinical chemotherapy, regardless of the mechanism, by virtue of exercising selective cytotoxicity has a target on which it acts [6], even if that target isn't known or understood. However, when advanced tumors are confronted with targeted or “untargeted” chemotherapies, alone or in combination, the result is almost always the same—acquired resistance.

Nevertheless, as targeted therapies [7] become the blueprint and the prevalent paradigm in drug development and clinical oncology, there is a danger that oncologists will overestimate the role of these therapies and, at the same time, undervalue the impact of their toxicity. Lamentably, because quality of life is not generally assessed in clinical trials as an approval endpoint, targeted chemotherapies in general cannot be properly evaluated from a benefit-toxicity perspective.

## METHODS AND RESULTS

By analogy with a survey in neuropsychology called MACH-IV by Christie and Geis in 1970 [1, 8], which measures Machiavellianism as a distinct personality construct, and ranks orientation according to high or low Mach, we have defined a rudimentary MACH scale for targeted chemotherapies, based on the means-to-ends ratio of toxicity and benefit, derived from a Phase 3 registration trial. The adverse event ratio is defined as the ratio of the percentage of Grade 3 and Grade 4 adverse events of the study drug to the comparator:

MACH Index = Adverse Event Ratio

### Survival ratio

Where the *Adverse Event Ratio* is the ratio of the percent of Grade 3 and higher adverse events for the study drug divided by percent of Grade 3 and higher adverse events for the comparator and the Survival ratio is the number of months of median OS for the study drug over the median OS for the comparator in months.

Although comparisons between different agents depend on the type of comparator in the registration trial, with the use of placebo potentially resulting in the highest MACH index, in general, the higher the MACH index the less desirable the agent, at least from a quality of life perspective. In this article we compare the MACH Index for the following small molecule inhibitors: regorafenib (Stivarga), sorafenib (Nexavar), bortezomib (Velcade), erlotinib (Tarceva), and sunitinib (Sutent) (Tables 1 & 2).

**Table 1:** Approved drugs and source data used in the analysis

Drug	Indication	Comparator	Trial name	Source data
Bortezomib	Multiple myeloma	Dexamethasone	APEX	Richardson, 2007^9,10^
Sunitinib	Metastatic Renal-Cell Carcinoma	Interferon alpha	-	Motzer, 2007^11^
Regorafenib	Metastatic colorectal cancer	Placebo	CORRECT	Grothey, 2012^12^
Sorafenib	Advanced Hepatocellular Carcinoma	Placebo	SHARP	Llovet, 2008^13^
Erlotinib	Advanced non-small-cell lung cancer	Placebo	SATURN	Coudert, 2012^14^

**Table 2:** Adverse event and survival ratios with MACH index for drug/comparator pairs

Drug/Comparator	Adverse Events	Adverse Event ratio	Survival (mo)	Survival ratio	MACH Index
Bortezomib	0.75	1.25	29.8	1.26	0.99
Dexamethasone	0.60		23.7		
Sunitinib	0.37	1.48	26.0	1.30	1.14
Interferon alpha	0.25		20.0		
Regorafenib	0.54	3.86	6.40	1.28	3.02
Placebo	0.14		5.00		
Sorafenib	0.11	2.06	10.7	1.28	1.62
Placebo	0.05		7.9		
Erlotinib	0.65	1.12	12.3	1.11	1.01
Placebo	0.58		11.1		

## DISCUSSION

The cardinal rule of medicine, primum non nocere, implied in the Hippocratic Oath, is inviolate except in the case of a life-threatening medical emergency such as cancer, in which case according to the unwritten elastic doctrine of Medical Machiavellianism the physician is authorized to take extraordinary measures to extend life, including the administration of systemically toxic conventional chemotherapy. This is justified by the scorched earth, win-at-all-costs ethos that has dominated the oncology landscape ever since President Richard Nixon launched the “War on Cancer” in 1971 [9, 10]. By contrast, the perception generally is that targeted therapy, being targeted, has a better safety profile, resulting in an improved functional status and health-related quality of life, and thereby allowing the clinician to adhere to a primum non nocere strategy. The reality [11] is different: many of the agents that were initially characterized as a ‘single blade’, specific for a particular gene or receptor are actually more akin to a Swiss Army knife, with multifunctional properties due to inhibition of more than one receptor, gene or pathway; the dark side of this promiscuous pharmacology is a new spectrum of ‘off-target’ side effects, distinct from conventional chemotherapy, which are sometimes severe, and therefore it is important to determine for *both* targeted and conventional chemotherapy whether the absolute magnitude of survival benefit is worth the added risks and toxicities since the only proven curative treatments are still surgery and radiotherapy.

Given the ubiquity of their use and uncertainty regarding the risk:benefit ratio, it is clearly time to examine targeted therapies in a careful and systematic way, emphasizing determinations of health-related quality of life (QOL), a concept defined as net satisfaction with life in the context of the cumulative risks and benefits of treatment, that are standard coin for medical decision-making in other disciplines [12]. PD-1 (programmed cell death protein 1) and CTLA-4 (cytotoxic T-lymphocyte-associated protein 4) immune checkpoint inhibitors may represent a new era and a paradigm shift in cancer therapy, but, even so, the consensus is that targeted agents are not only here to stay [19] but will continue to play a significant role in combination with chemo and immunotherapy.

**Figure 1:**
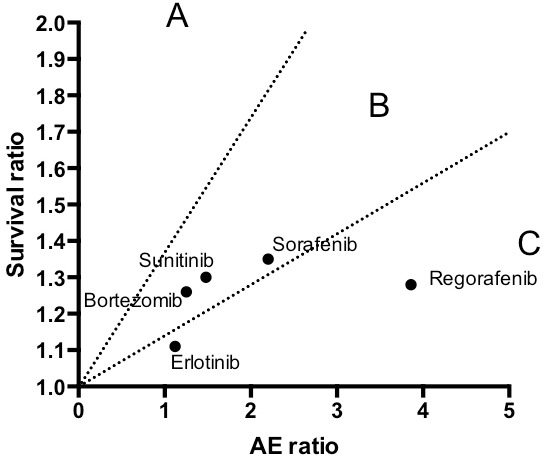
Survival and adverse event ratios of selected targeted anti-cancer drugs Regions are defined as: **A.** Favorable high survival benefit to low toxicity;. **B.** Balance survival vs. toxicity; **C.** unfavorable survival benefit to toxicity.

Our analysis of targeted therapies allows some insight into their relative risk to benefit ratio. Ideal anti-cancer therapies, that is those that prolong survival with minimal adverse events, would fall into the region defined as ‘A’ in Figure 1, while therapies that provide limited benefit with a high rate of therapy-related adverse events fall into region ‘C’. Unfortunately, none of the five targeted therapies studied fall into the high benefit-low morbidity region ‘A’. The majority of the profiled drugs cluster in the intermediate region ‘B’ of the graph, occupying a middle ground between toxicity and benefit. At the other end of the spectrum from region ‘A’, regorafenib and erlotinib are found in region ‘C’ where toxicity predominates over clinical benefit, suggesting that these agents should be prescribed with special caution.

## CONCLUSIONS

While Figure 1 may indicate that our understanding of the genetic vulnerabilities and dependencies of cancer has not advanced sufficiently to permit development of highly effective molecularly targeted agents with non-toxic safety profiles, it is anticipated that a new generation of bellwether onco-immunology agents, from checkpoint inhibitors to oncolytic viruses, currently in development, will usher in a paradigm shift toward region ‘A’ of the graph.

Although our approach is quite plainly *ad hoc*, subjective, and heavily dependent on the particular disease under investigation as well as the comparator in the clinical trial, we hope the intention to benchmark these ‘targeted’ agents with an easily accessible rating system will serve to highlight the centrality of QOL as a primary index for individual patients rather than relegating it to the all-too-familiar status of mere window dressing or icing on the cake after overall survival.

Finally, while it may seem somewhat shocking and heretical to associate the practice of oncology, an ostensible ethical and moral good, with Machiavellian tactics, the fact is that in the face of metastatic cancer the absolute standard of non-malfeasance—“do no harm”—does not necessarily apply and tough tradeoff treatment decisions are frequently necessary to extend survival. In this context, it is our hope that this article may help guide oncologists, in true Machiavellian fashion, to choose the lesser of two necessary targeted evils.

## Abbreviations

CTLA-4: Cytotoxic T-lymphocyte-associated protein 4
MM: Medical Machiavellianism
OS: Overall survival
PD-1: Programmed cell death protein 1
QOL: Quality of Life
VEGFR2: vascular endothelial growth factor receptor 2
